# EGFR Signaling Promotes β-Cell Proliferation and Survivin Expression during Pregnancy

**DOI:** 10.1371/journal.pone.0093651

**Published:** 2014-04-02

**Authors:** Elina Hakonen, Jarkko Ustinov, Jaan Palgi, Päivi J. Miettinen, Timo Otonkoski

**Affiliations:** 1 Research Programs Unit, Molecular Neurology, Biomedicum Stem Cell Center, University of Helsinki, Helsinki, Finland; 2 Children's Hospital, University of Helsinki and Helsinki University Central Hospital, Helsinki, Finland; University of Bremen, Germany

## Abstract

Placental lactogen (PL) induced serotonergic signaling is essential for gestational β-cell mass expansion. We have previously shown that intact Epidermal growth factor –receptor (EGFR) function is a crucial component of this pathway. We now explored more specifically the link between EGFR and pregnancy-induced β-cell mass compensation. Islets were isolated from wild-type and β-cell-specific EGFR-dominant negative mice (E1-DN), stimulated with PL and analyzed for β-cell proliferation and expression of genes involved in gestational β-cell growth. β-cell mass dynamics were analyzed both with traditional morphometrical methods and three-dimensional optical projection tomography (OPT) of whole-mount insulin-stained pancreata. Insulin-positive volume analyzed with OPT increased 1.4-fold at gestational day 18.5 (GD18.5) when compared to non-pregnant mice. Number of islets peaked by GD13.5 (680 *vs* 1134 islets per pancreas, non-pregnant *vs*. GD13.5). PL stimulated beta cell proliferation in the wild-type islets, whereas the proliferative response was absent in the E1-DN mouse islets. Serotonin synthesizing enzymes were upregulated similarly in both the wild-type and E1-DN mice. However, while survivin (*Birc5*) mRNA was upregulated 5.5-fold during pregnancy in the wild-type islets, no change was seen in the E1-DN pregnant islets. PL induced survivin expression also in isolated islets and this was blocked by EGFR inhibitor gefitinib, mTOR inhibitor rapamycin and MEK inhibitor PD0325901. Our 3D-volumetric analysis of β-cell mass expansion during murine pregnancy revealed that islet number increases during pregnancy. In addition, our results suggest that EGFR signaling is required for lactogen-induced survivin expression via MAPK and mTOR pathways.

## Introduction

Pregnancy is one of the most robust physiological stimuli of β-cell mass expansion in the adult pancreas. This compensates for the rise in insulin demand, which is due to reduced insulin sensitivity in target tissues. In pregnant rodents, β-cell proliferation increases dramatically, with a peak occurring after mid gestation and returning to postpartum levels after day 18.5 [1], [2]. The most important factors leading to β-cell mass expansion during pregnancy are the lactogenic hormones prolactin (Prl) and placental lactogen (PL) which both activate the prolactin receptor (Prl-R) [3]. The peak in β-cell proliferation coincides with the peak of PL levels [1]. Ligand-binding to the Prl-R leads to activation of the janus kinase 2 (JAK2)-signal transducer and STAT5 pathways [4]. In addition, Prl-R can activate MAPK and PI3K pathways [5]. Several studies have explored the gene network changes in islets during pregnancy [6]–[8]. Placental lactogen controls β-cell mass expansion at multiple levels, including upregulation of serotonin synthesis by β-cells [7], [9], induction of the transcription factor FoxM1 [10], downregulation of Menin [11], and upregulation of survivin (*Birc5*) [6].

Many studies have shown that increased proliferation of β-cells is the key mechanism explaining the expansion of β-cell mass during pregnancy [2], [10], [12], [13]. It has been suggested that the number of islets remains constant whereas individual islets grow in size [14]. On the other hand, some studies report that in addition to increased proliferation, the number of islets increases during pregnancy [3]. Supporting this, a lineage-tracing experiment by Abouna et al. shows that some non-β-cells differentiate into insulin-producing cells during pregnancy suggesting islet neogenesis [15]. Furthermore, autopsy studies of human maternal pancreata during pregnancy have shown an increase in the number of small islets rather than increased β-cell proliferation [16].

The measurement of β-cell mass and islet number has so far been mostly based on approximation from two-dimensional models based on insulin immunohistochemistry. Optical projection tomography (OPT) was recently introduced for reliable visualization of β-cell mass in the intact pancreas [17], [18]. This method allows three-dimensional imaging of insulin-positive islets from the whole pancreas and quantification of insulin-positive volume as well as islet number. OPT has not been previously used to measure pregnancy-induced islet mass expansion.

We have previously shown that EGFR signaling is important in pancreatic development, survival and postnatal β-cell proliferation, particularly during pregnancy [12], [19], [20]. In this study we investigated in more detail the molecular mechanisms through which EGFR participates in gestational β-cell compensation and have applied OPT for the 3D-analysis of islet number and β-cell mass during pregnancy.

## Materials and Methods

### Animals

The E1-DN mice were generated as previously described [19]. Briefly, the E1-DN transgene consists of the mouse Pdx1 promoter, followed by a betaglobin second intron, the human kinase-deficient EGFR cDNA with a myc-tag and a growth hormone polyA tail. All mice were maintained in FVB background, housed on a 12-h light-dark cycle and fed ad libitum without restriction to physical activity. The study protocol was approved by The National Animal Experiment Board of Finland (permit number ESAVI-2010-09238/Ym-23). Wild-type controls were derived from litters or non-related litters. For the pregnancy experiment, E1-DN and wild-type mice were mated with male FVB mice. Mating was confirmed by the presence of a vaginal plug on the next morning, designated as day 0.5 of gestation. Mice were killed with CO2 >70% followed by cervical dislocation and samples collected on days 13.5, 14.5 and 18.5 of pregnancy.

### Immunohistochemistry

The paraffin sections were stained as previously described [12]. Antibodies used were guinea pig anti-swine insulin (Dako Cytomation, Glostrup, Denmark), mouse anti-BrdU (Dako Cytomation), rabbit anti-survivin (Cell Signalling Technology), rabbit anti-C-peptide (Cell Signalling Technology, Danvers, MA, USA) and rabbit anti-ChromograninA (Dako Cytomation). Specificity of the survivin antiserum was confirmed by preabsorbtion with a survivin blocking peptide for 30 min (Cell Signalling Technology).

For light microscopy stainings the sections were incubated for 30 min at room temperature with a biotinylated secondary antibody (Zymed Laboratories, South San Francisco, CA, USA), rinsed and incubated with peroxidase-conjugated streptavidin (Zymed Laboratories). The sections were finally developed with 3-amino-9-ethyl-carbazole substrate (Thermo Scientific). For fluorescence microscopy, the secondary antibodies used were goat anti-guinea pig IgG (Alexa, A11076, Invitrogen, Paisley, UK), donkey anti-rabbit IgG (Alexa, A21206, Invitrogen) and donkey anti-mouse IgG (Alexa, A21202, Invitrogen). Nuclear staining was performed with DAPI (Vectashield with DAPI, Vector Laboratories, Burlingame, CA, USA).

### Organ preparation and OPT scanning

The pancreata of FVB control and pregnant mice were processed as described by Alanentalo et al. [17]. Shortly, the pancreata were fixed in 4% PFA for 4 h, dehydrated in methanol, quenched in MeOH: DMSO: H_2_O_2_ (2∶1∶3), followed by rehydration to TBST and incubations with guinea pig anti-swine insulin (DakoCytomation, Glostrup, Denmark) and goat anti-guinea pig IgG (Alexa, A11076, Invitrogen). The stained pancreata were mounted in low melting agarose, followed by dehydration in methanol and clearing with BABB. Bioptonics 3001 OPT scanner (Bioptonics) was used for the scanning of the samples, with exiter D560/40 nm and emitter 610 nmLP or exiter 425/40 nm and emitter 475 nmLP when visualizing Alexa 594 and 488 respectively. Scanning settings (identical for all specimen) were: rotation degree 0,9 um; pixel size 18,23 um; resolution 1024×1024 pixels; exposure time was set manually depending of the intensity of the fluorescence. All samples were scanned with the same zoom. OPT sample reconstructions were made with NRecon v1.6.3.3 (SkyScan). Isosurface reconstructions were generated using Imaris v 7.6.3 (Bitplane). Islet volumes were segmented using the “background subtraction (local contrast)” thresholding option and the intensity threshold was set manually for each pancreas.

### 2D Beta cell mass and islet size distribution analysis

For the morphometrical beta cell mass quantification, pancreata were either fixed directly in 4% PFA o/n and processed with routine methods or fixed and processed as described above for the OPT analysis and after OPT imaging washed in methanol, rehydrated in ethanol series and incubated in 0.29 M sucrose to remove the agarose followed by routine processing to generate paraffin blocks. The pancreatic paraffin blocks were sectioned and five sections, one per 100–150 μm intervals, were analysed. These sections were stained for insulin, counterstained with haematoxylin and morphometrically analysed directly under light microscopy using the Image-Pro Analyser 6.0 software (Media Cybernetics, Bethesda, MD). The beta cell mass was calculated by multiplying the relative cross-sectional area of the insulin-positive area per total pancreatic tissue area by the weight of the pancreas. Islets were classified into small <12076 μm^2^, medium 12076–35033 μm^2^ and large islets >35033 μm^2^ and the amount of islets was calculated as relative to total pancreatic area and further corrected by multiplying with the pancreatic weight.

### Islet isolation

Islets from wild-type and E1-DN control and pregnant mice were isolated by collagenase digestion (Collagenase P; Roche diagnostics, Mannheim, Germany) followed by handpicking under a stereomicroscope. Directly after isolation the islets were lysed in the RNA extraction buffer (NucleoSpin RNAII kit, Macherey-Nagel, Düren, Germany). For proliferation analysis and PL stimulation experiments islets were cultured as described below.

### Quantitative RT-PCR

Total RNA from wild-type and E1-DN islets was isolated using the NucleoSpin RNAII kit (Macherey-Nagel) without on-column DNase treatment. DNase treatment was done separately followed by RNA purification with NucleoSpin RNA CleanUp XS kit according to manufacturer's instructions (Macherey-Nagel). RNA quality was controlled with NanoDrop 1000 spectrophotometer (Thermo Scientific, Wilmington, DE, USA). cDNA was synthesized from approximately 70 ng of islet RNA. Real-time PCR was done with SYBR Green JumpStart Taq ReadyMix for Quantitative PCR (Sigma-Aldrich, ST Louis, MO, USA) with Corbett Rotor-Gene 6000 instrument (Qiagen, Hilden, Germany). The reactions were prepared with a Corbett CAS-1200 liquid handling system. All reactions were performed in duplicates on at least three biological replicates. The median C_T_ values were used for ΔΔCT analysis [21] with housekeeping gene Cyclophilin G as endogenous control. Exogenous positive control was used as a calibrator for all the real-time PCRs. Primer sequences are presented in Table S1. Human and endogenous mouse transcripts were distinguished with species-specific primers (Table S1). DNA extraction and genotyping of the mice were done as previously described by Miettinen et al [19].

### Cell culture

In proliferation experiments mouse islets were cultured in RPMI supplemented with Glutamax, penicillin (100 IU/ml), streptomycin (100 IU/ml) and 10% fetal calf serum either with or without PL 500 ng/ml (Affiland, Liège, Belgium) for 96 h. BrdU 30 μg/ml (Sigma-Aldrich) was added for the last 48 h. For RNA isolation the islets were cultured in RPMI supplemented with Glutamax, penicillin (100 IU/ml), streptomycin (100 IU/ml), 10% fetal calf serum and stimulated with 500 ng/ml PL (Affiland), 2 μM gefitinib (Selleckchem, Houston, TX, USA), 10 nM rapamycin (Selleckchem) or 0,5 μM MEK inhibitor PD0325901 (Selleckchem) for 96 h. All inhibitors were dissolved in DMSO and DMSO 0,02% was also added to the control and PL only media. After stimulation the islets were lysed in the RNA extraction buffer (NucleoSpin RNAII kit).

### Statistical analysis

All data are presented as means ±SEM from at least three independent experiments. Significances of the differences between three or more groups were calculated with one-way ANOVA followed by Tukey's test, using Graph Pad Prism 6 software.

## Results

### β-cell mass of E1-DN mice does not grow during pregnancy

To investigate the molecular mechanism by which EGFR signaling participates in beta cell proliferation during pregnancy, E1-DN mice were used as a model. These mice express a mutant kinase-negative EGFR under the Pdx1 promoter, which leads to approximately 40% reduction in EGFR phosphorylation upon EGF stimulation in homozygous animals. In heterozygous animals, which were used in these experiments, the phenotype is milder and the mice are normoglycemic under normal physiological situations [12].

**Figure 1:**
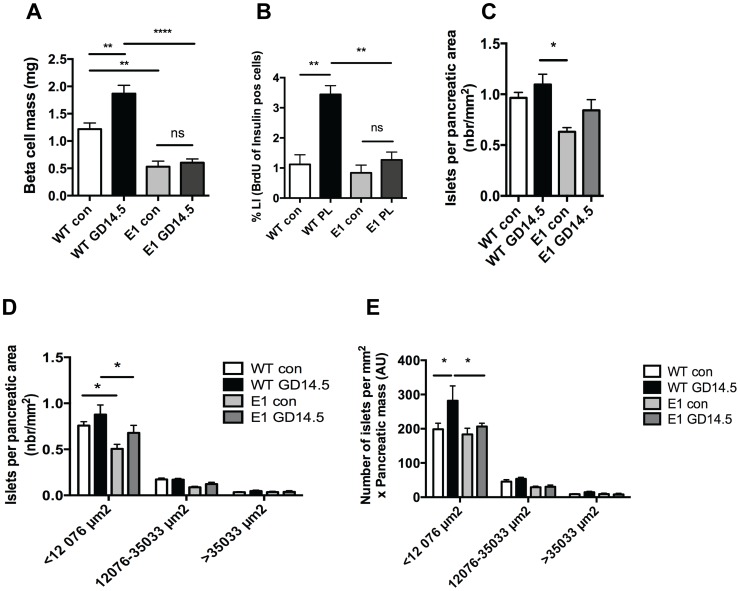
E1-DN mice do not increase β-cell mass during pregnancy. A: The β-cell mass of wild-type mice increases during pregnancy at GD14.5. There is no difference between groups of E1-DN mice, n = 8–13. B: Isolated islets from wild-type (WT) and E1-DN (E1) female virgin mice were cultured for 96 h either with PL 500 ng/ml or in control media (con). BrdU was added to the media for the last 48 h. Quantification of proliferative β-cells, n = 3 in each group. C: Total number of islets per pancreatic area from WT and E1-DN (E1) control and pregnant gestational day 14.5 (GD14.5) mice, n = 4–6 per group. D: The distribution of islet sizes relative to total pancreatic area. Islets were classified into small <12076 μm^2^, medium 12076–35033 μm^2^ and large islets >35033 μm^2^ from wild-type (WT) and E1-DN (E1) control (con) and gestational day 14.5 (GD14.5) pancreata, n = 4–6 per group. E: The islet size distribution corrected for pancreatic weight. AU, arbitrary unit. Bars represent the mean ±SEM for each group. *p<0.05, **p<0.01,***p<0.001, ****p<0.0001 ns = no statistical significance.

During pregnancy the β-cell mass of wild-type mice increased by 50% (from 1,2 to 1,8 mg, p<0.01). In contrast, the E1-DN mice did not show any increase in β-cell mass while pregnant (0,5 vs 0,6 mg, ns) (Fig 1A). We have previously shown that *in vivo* the proliferation of E1-DN β-cells does not increase during pregnancy [12]. However, it was unclear whether the reduced proliferation is due to a defective effect of PL/Prl action on beta cells or due to a reduced proliferative effect of the EGFR ligands. To elucidate this, we isolated islets from wild-type and E1-DN virgin mice, stimulated them *in vitro* for 96 h with PL and analyzed β-cell proliferation rate. Indeed, we could detect an increase in the proliferation of wild-type β-cells (from 1% to 3,5%) but this increase was absent from the E1-DN mouse islets correlating with the *in vivo* results (Fig 1 B).

### Quantitative morphometry of islets during pregnancy

In addition to β-cell proliferation, some studies report that also the number of islets increases during pregnancy. To investigate the effect of EGFR signaling on the possible formation of new islets during pregnancy we analyzed the number of islets per pancreatic area from non-pregnant mice and at gestational day 14.5 (GD14.5). The wild-type mice had more small islets than the E1-DN mice in both pregnant and non-pregnant pancreata (Fig 1C–D). Additionally, there was a trend for increased islet density during pregnancy in both wild-type and E1-DN mice, especially in small islets (Fig 1C–D) suggestive of islet neogenesis. When the islet density was multiplied by pancreatic weight, there was a significant increase in the number small islets in the wild-type mice (Fig 1E).

In classical two-dimensional stereological analysis the whole pancreas is sectioned and examined at repeated intervals to cover the entire depth of the pancreas. However, the quantification of the total islet number is challenging. New three-dimensional (3D) methods enable the quantification of insulin-positive volume from the whole pancreas, providing also information about the number of individual islets in addition to total insulin-positive volume. To further clarify the possible formation of new islets during pregnancy and to study the dynamics of β-cell mass changes in 3D, we applied the three-dimensional OPT method to wild-type control and pregnant mice. Two time-points were chosen: gestational day 13.5 (GD13.5), when β-cell proliferation has been shown to peak, and day 18.5 (GD18.5) when the β-cell mass is at its highest [13].

**Figure 2:**
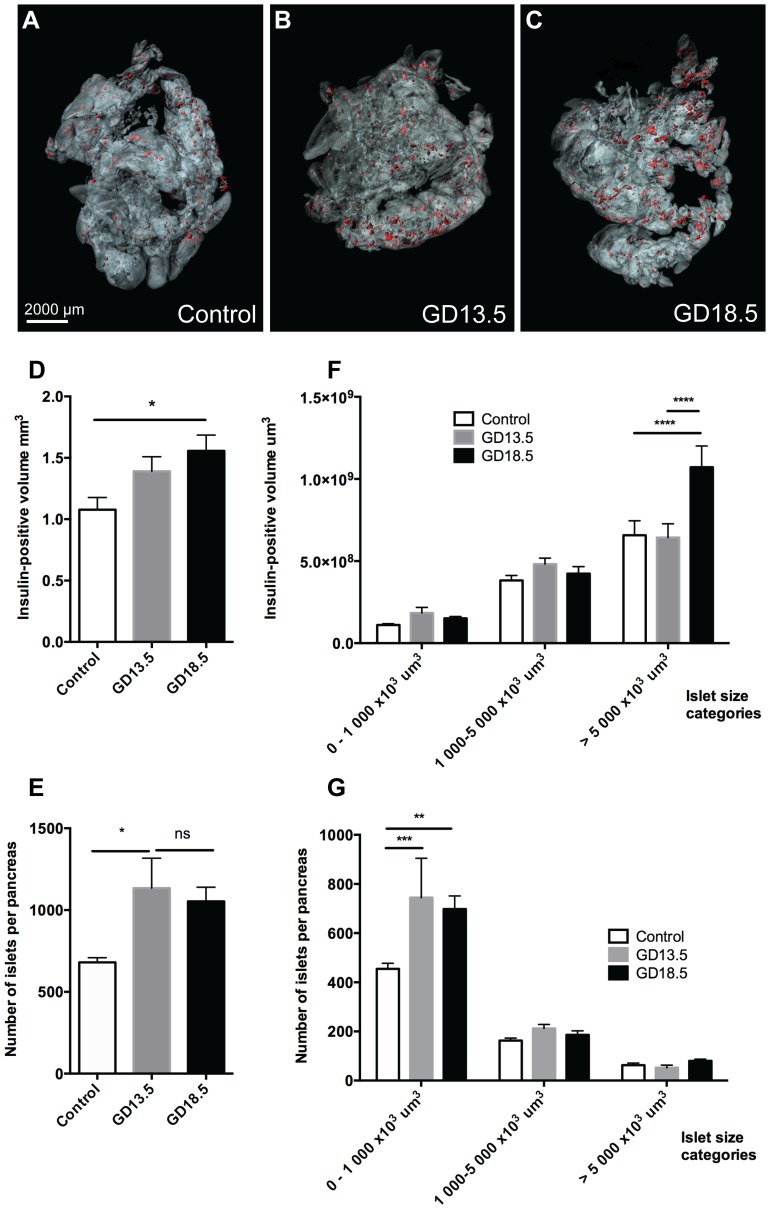
OPT analysis of insulin-positive volume and islet number during pregnancy. A–C: Isosurface rendered OPT images of insulin labeled (red) representative pancreata from control (A), gestational day 13.5 (GD13.5) (B) and gestational day 18.5 (GD18.5) (C) female mice. The pancreas outline is based on autofluorescence of the tissue. D: Graph illustrating the average insulin-positive volume in control (white), GD13.5 (gray) and GD18.5 (black) pancreata, n = 7–8 per group. E: Graph illustrating the average number of islets per pancreas in control (white), GD13.5 (gray) and GD18.5 (black) pancreata, n = 7–8 per group. F–G: The distribution of islet sizes (G) and contribution to total islet volume (F) n = 7–8 per group. Islets were classified into small 0–1000 um^3^×10^3^, intermediate 1000–5000 um^3^×10^3^ and large islets >5000 um^3^×10^3^ from control (white), GD13.5 (gray) and GD18.5 (black) pancreata. Bars represent the mean ±SEM for each group. *p<0.05, **p<0.01, ***p<0.001 ns = no statistical significance.

With the three-dimensional approach, we detected a 1.2-fold increase in the insulin-positive volume at GD13.5 and a 1.4-fold increase by GD18.5 (Fig 2 A–D, Movie S1). Interestingly, the islet number increased by GD13.5 (from 680 to 1134 islets per pancreas, p<0.001) but remained steady from GD13.5 to GD18.5 (1134 vs 1054 islets, ns) (Fig 2E). The increase in islet number was mainly due to increase in the smaller islets (0–1000×10^3^ μm^3^; Fig 2G). However, large islets accounted for most of the total β-cell mass and also during pregnancy, the absolute increase in the β-cell volume was mostly due to the increased volume of the large (>5×10^6^ μm^3^) islets (Fig 2F).

**Figure 3:**
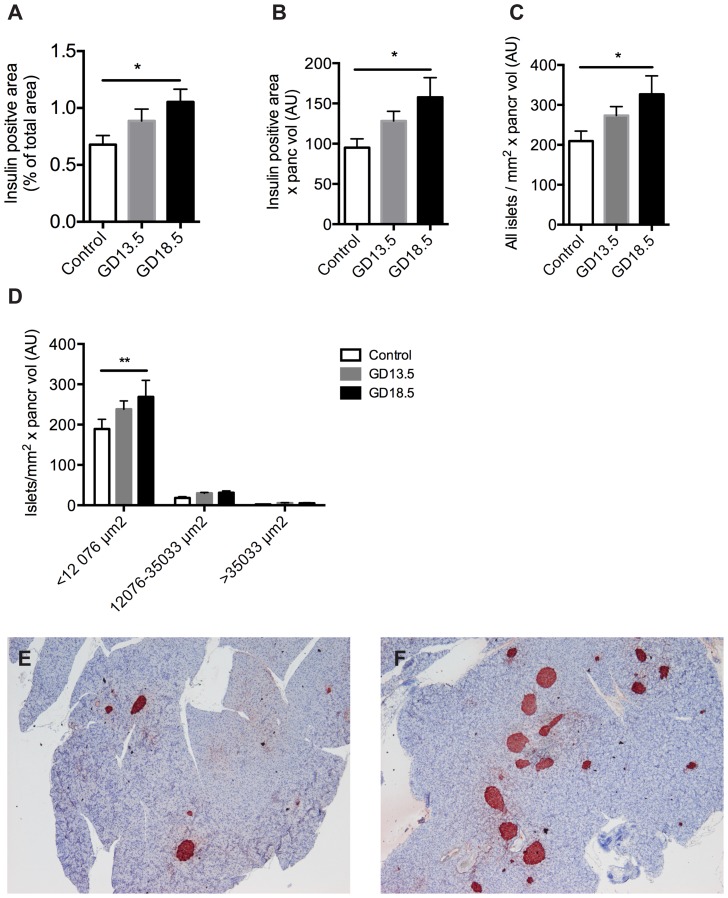
Validation of OPT method by 2D morphometrical analysis. The same pancreata that were analyzed by OPT were sectioned and analyzed with routine methods. A: Insulin positive area per total pancreatic area. B: Insulin positive surface area per pancreatic area multiplied by total pancreatic volume. C: Number of islets per pancreatic area multiplied by total pancreatic volume. D: Islets were classified into small <12076 μm^2^, medium 12076–35033 μm^2^ and large islets >35033 μm^2^ and the distribution was calculated per total surface area and further multiplied by total pancreatic weight. AU, arbitrary unit. Bars represent the mean ±SEM for each group, n = 6–8. *p<0.05, **p<0.01. E–F: Representative images of insulin immunohistochemistry (red) from non-pregnant (E) and pregnant GD18.5 (F) pancreas.

In order to directly compare the islet number and beta cell mass results between the OPT and classical morphometric 2D method, we sectioned the same pancreata that were analyzed by OPT and subjected them to the 2D analysis. This method revealed a 34% increase in insulin-positive volume by GD13.5 and a 53% increase by GD18.5 (Fig 3 A–B). The proportional increase in small islets by GD13.5 was less than obtained with OPT (Fig 3 C–E). However, by GD18.5 the number of small islets had increased significantly also with this method (Fig 3D–F).

### Survivin expression is not increased in E1-DN islets during pregnancy

**Figure 4:**
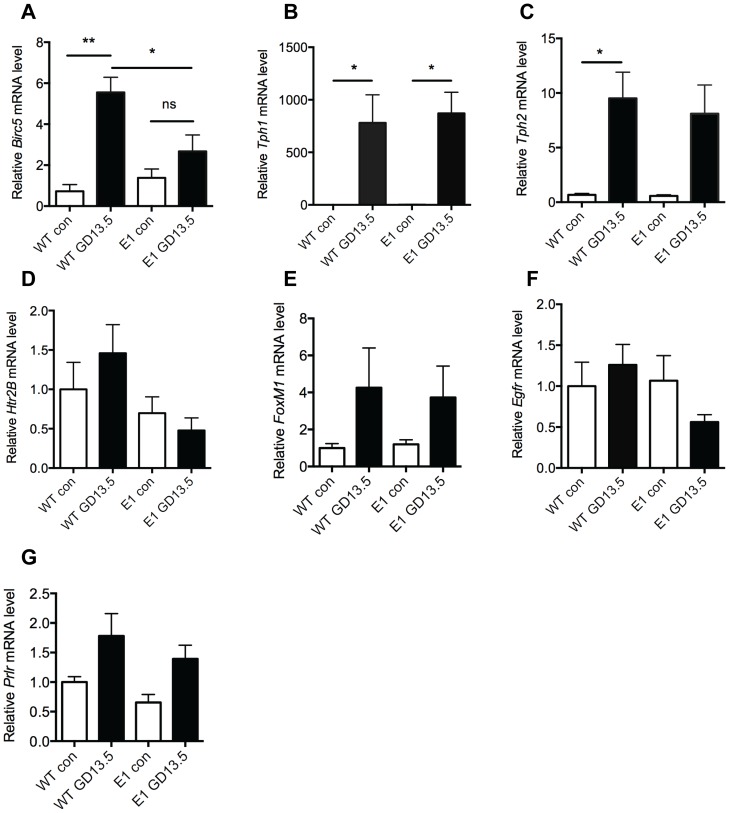
Reduced EGFR signaling leads to decreased survivin expression in islets during pregnancy in mice. A–G: Islets were isolated from wild-type (WT) and E1-DN (E1) virgin (con) and gestational day 13.5 (GD13.5) mice and RT-qPCR performed. Graphs are showing the relative mRNA level of survivin (A), *Tph1* (B), *Tph2* (C), serotonin receptor *Htr2B* (D), *FoxM1* (E), endogenous mouse *Egfr* (F) and prolactin receptor (G) n = 4–5 per group. *p<0.05, **p<0.01, ns = no statistical significance.

Next we investigated why the E1-DN islets do not respond to lactogenic stimulation. We isolated islets from maternal pancreata at GD13.5 from wild-type and E1-DN mice and analyzed the expression of selected genes. Survivin has been shown to be upregulated during pregnancy and is a known target for EGFR signaling [6], [22]. Interestingly, survivin (*Birc5*) expression was highly (up to five-fold) upregulated in wild-type islets during pregnancy, but this increase was lacking from the E1-DN islets (Fig 4A). Additionally we analyzed expression of the serotonin-synthesizing enzymes *Tph1* and *Tph2* and the Serotonin receptor *Htr2B*, which all have been shown to be involved in the regulation of gestational β-cell proliferation [7], [9]. *Tph1* and *Tph2* were upregulated at a similar level in both the wild-type and E1-DN islets (Fig 4B–C). Furthermore, there was no difference in *Htr2B* expression level between the groups (Fig 4 D). We also analyzed the expression of *FoxM1*, which has been shown to be required for beta cell proliferation during pregnancy [10]. No difference could be detected in *FoxM1* expression between the genotypes (Fig 4 E). The human *EGFR* transgene was abundantly expressed in the E1-DN mice as expected (data not shown), while the endogenous *Egfr* expression did not significantly change between the genotypes or during pregnancy (Fig 4F). Finally, there was no difference in the *Prlr* expression during pregnancy between the genotypes (Fig 4 G).

**Figure 5:**
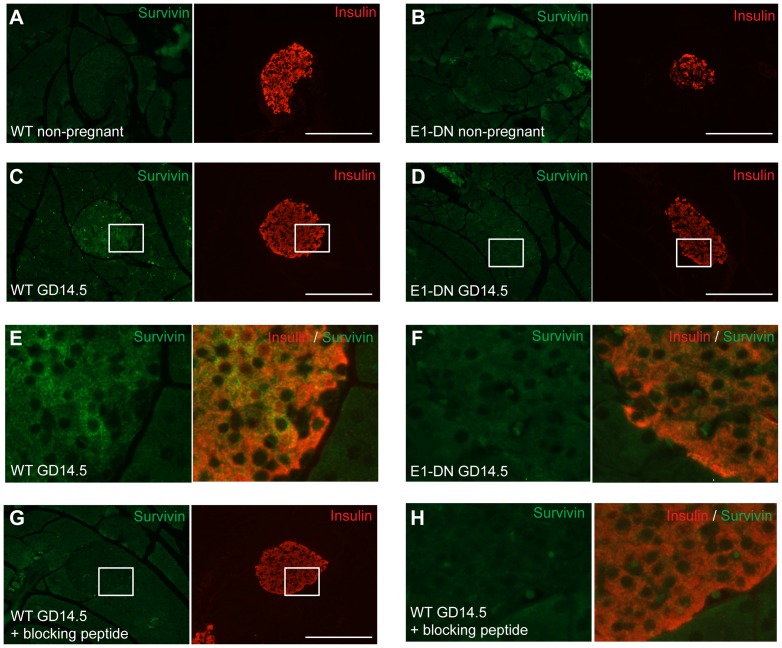
Survivin immunoreactivity is detectable in islets of pregnant wild-type but not E1-DN mice. A–H: Double immunohistochemistry for survivin (green) and insulin (red) from wild-type non-pregnant (A), E1-DN non-pregnant (B), wild-type pregnant GD14.5 (C) and E1-DN pregnant GD14.5 (D) mouse. Scale bar 200 μm. E: Magnification of squared area in C. F: Magnification of squared area in D. G: Negative control from wild-type pregnant mouse pancreas with blocking peptide. Scale bar 200 μm. H: Magnification of squared area in G.

The difference in survivin expression was confirmed at protein level by immunohistochemistry, since survivin-like immunoreactivity was clearly detectable in the β-cells of the pregnant wild-type mice, while only a very faint signal was obtained in the islets of the pregnant E1-DN mice (Fig 5 A–H). Preabsorption of the anti-survivin with a blocking peptide abolished completely the fluorescence serving as a specificity control.

### Survivin upregulation depends on EGFR, mTOR and MEK

**Figure 6:**
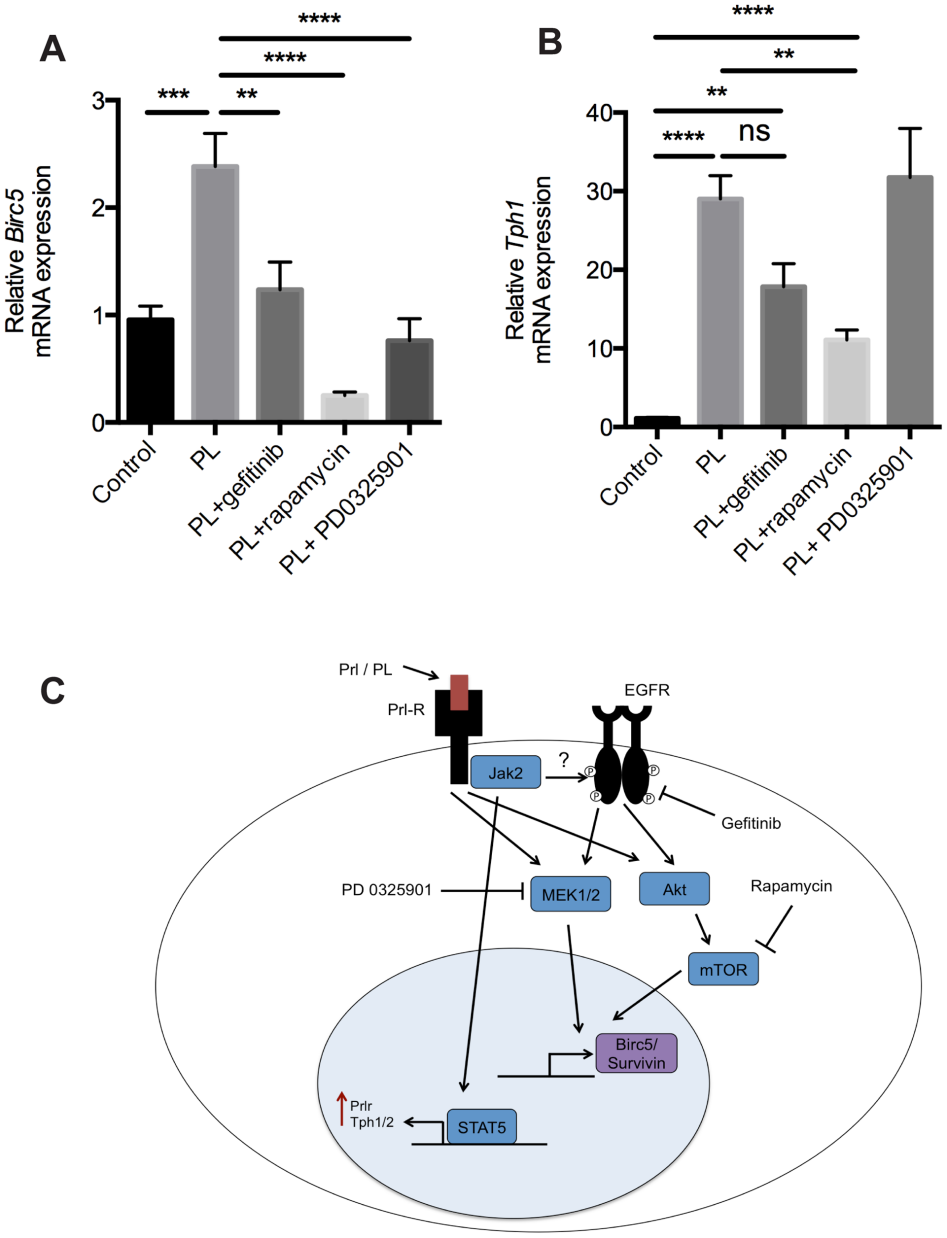
PL-induced survivin upregulation is dependent of EGFR, mTOR and MEK. A–B: Islets were isolated from wild-type mice, stimulated with or without PL (500 ng/ml), gefitinib (2 μM), rapamycin (10 nM), MEK inhibitor PD0325901 (0,5 μM) or EGF (50 ng/ml) and BTC (50 ng/ml) for 96 h and RT-qPCR performed. Graph is showing the relative mRNA level of survivin (A) and *Tph1* (B). n = 6–9 per group. Bars represent the mean ±SEM for each group. *p<0.05, **p<0.01 ***p<0.001. C: Proposed model for PL-induced survivin upregulation. Prl-R activation leads to transactivation of EGFR, which activates MAPK and PI3K-Akt-mTOR pathways, that together stimulate survivin gene expression.

To determine whether PL stimulation induced survivin upregulation could be blocked by EGFR inhibitor *in vitro* and which signaling pathways are involved, we isolated pancreatic islets and stimulated them with PL with or without EGFR inhibitor gefitinib or MEK inhibitor PD0325901 or mTOR inhibitor rapamycin for 4 days. PL induced a 2.3-fold increase in survivin mRNA expression and this increase was blocked by gefitinib and by MEK inhibitor as well as by rapamycin (Fig 6 A). On the contrary, PL-induced *Tph1* upregulation, which is stimulated by JAK2/STAT5 pathway, could not be blocked by gefinitib nor MEK inhibitor, as expected (Fig 6 B). PL-induced *Tph1* upregulation was reduced by rapamycin when compared to PL alone, but the expression level remained significantly higher than in the controls (Fig 6B).

## Discussion

EGFR signaling has been linked to both β-cell replication and differentiation [23]–[26]. We have previously shown that the EGFR is crucial in pregnancy-induced β-cell mass expansion, primarily due to inadequate β-cell proliferation of the maternal islets [12]. In this study we aimed to investigate in more detail the molecular mechanism linking EGFR signaling with beta cell proliferation during pregnancy. We took advantage of the beta-cell specific EGFR dominant negative mouse model which we created earlier (the E1-DN mice) [19]. The heterozygous mice, which were used in these experiments, are euglycemic under normal physiological conditions. However, the mice are unable to increase their beta cell mass during pregnancy and become hyperglycemic [12].

In addition to direct ligand binding, the ErbB receptors have been shown to act as signal integrators, as they can act as targets of signaling events emanating from other receptors [27], [28]. Since lactogens are the most important stimulators of β-cell mass expansion during pregnancy, we hypothesized that Prl-R signaling acts partially through EGFR. The involvement of EGFR in Prl-R signaling is supported by previous findings of the interplay between EGF and Prl in breast cancer cells. Prl induces threonine phosphorylation of EGFR through ERK pathway and synergistically augments EGF signaling by inhibiting the downregulation of activated EGFR [29], [30]. In our study we show that the PL-induced β-cell proliferation was affected by downregulation of EGFR signaling in murine islets. When E1 islets were stimulated with PL *in vitro*, the β-cells could not increase their proliferation unlike the wild-type β-cells.

In addition to increased β-cell proliferation, several studies have reported formation of new islets during pregnancy [3], [15], [16]. On the other hand, some studies argue against islet neogenesis during pregnancy [14]. To explore whether EGFR signaling also affects new islet formation and whether new islets actually do form, we analyzed islet number and islet size distribution from wild-type and E1-DN pancreata. Our data from 2D analysis implied that new islets were formed during pregnancy. This was further analyzed with the 3D OPT method. The results revealed that β-cell mass increased by 1.2-fold at GD13.5 and continued to expand until late pregnancy up to 1.4-fold when compared with the non-pregnant controls. Since OPT is a novel method and has not been previously used to study beta cell mass adaptation during pregnancy, we validated the method by doing also a 2D analysis from the same pancreata. In the 2D analysis the total beta cell volume was increased by 34% at GD 13.5 and by 53% at G18.5. These results are in line with the OPT results, although the magnitude of beta cell mass increase is slightly smaller with the OPT.

Interestingly, we could detect a prominent increase in the number of islets during pregnancy in the wild-type mice with both methods suggestive of islet neogenesis. However, the majority of the increase in islet volume in our data is due to increased volume of the largest islets, rather than by formation of new islets during pregnancy. Major weakness of the OPT analysis is that its detection limit excludes very small islets, i.e. less than 5×10^3^ um^3^ in size. Consequently, single β-cell or small β-cell clusters that fall below OPT detection limit may proliferate and increase in size above the detection limit and probably account for a part of the “new” islets. However, we rarely detect small islets with proliferating β-cells and the absolute increase in our study was up to 400 islets, making it unlikely that all the new islets would have resulted from the proliferation of existing small clusters. Furthermore, we did detect increased number of small islets also with the 2D morphometrical analysis. Lineage tracing approaches would be needed to confirm whether islet neogenesis actually occurs and from what cell type.

To elucidate the molecular mechanisms through which EGFR affects pregnancy-induced β-cell proliferation we isolated islets from wild-type and E1-DN pregnant and control mice and studied gene expression of selected genes (i.e. *Htr2B, Tph1* and *2, Birc5, FoxM1* and *Prlr*). During pregnancy the β-cells start to synthesize serotonin, which then acts in a paracrine-autocrine fashion through Htr2B to stimulate β-cell proliferation [9]. It has been shown that blocking Htr2B signaling during pregnancy also blocks β-cell mass expansion. However, there are controversial results concerning *Htr2B* expression levels during pregnancy, as Kim et al report that the receptor expression increases during pregnancy [9], while Schraenen et al did not see this [7]. Our results are in line with the latter result, showing no major changes in *Htr2B* expression during pregnancy.

The major difference between wild-type and E1-DN mice was that survivin was strongly upregulated during pregnancy in wild-type islets, while no upregulation was seen in the E1-DN islets. Survivin acts as an inhibitor of apoptosis that is also involved in cell division. It can inhibit both the intrinsic and extrinsic apoptosis pathways by blocking the activity of several caspase proteins [31], [32]. In addition, survivin forms complexes with chromosomal passenger proteins including aurora B kinase, INCENP and Borealin to regulate cell division [32]. In pancreas survivin is expressed during embryonic period throughout the pancreatic epithelium, but becomes gradually restricted to pancreatic β-cells. Survivin expression is very low in adult mouse islets but becomes upregulated during pregnancy and after pancreatic duct ligation [6], [33]. Mice lacking survivin in β-cells develop insulin-deficient diabetes after birth due to a failure of β-cell mass expansion [34], [35] and show defective β-cell mass expansion and proliferation after pancreatic injury [33]. On the other hand, overexpression of survivin does not induce β-cell proliferation but improves survival of islet grafts [36].

EGFR signaling has been linked to survivin in many cancer studies [37], and it has been shown that EGFR activates the PI3K/Akt pathway leading to upregulation of HIF-1alpha which subsequently activates survivin gene expression by binding to its promoter region [22]. In our study, survivin expression was upregulated during pregnancy in the maternal rodent islets and this increase was dependent of intact EGFR signaling, since no survivin upregulation was evident in the E1-DN pregnant islets. Importantly, we also detected survivin at the protein level only from wild-type islets during pregnancy, not from E1-DN pregnant pancreata.

*In vitro* stimulation of pancreatic islets with PL resulted in increased survivin expression. This effect was blocked by the tyrosine kinase inhibitor of EGFR, confirming that EGFR activation is needed for PL-induced survivin upregulation. EGFR activation in pancreatic beta cells leads to activation of MAPK and PI3K pathways [26], [38], [39]. Recently it was demonstrated that mTOR participates in EGFR signaling in beta cells during glucose and intralipid infusion induced insulin resistance, where the compensatory beta cell mass expansion was stimulated via the EGFR-PI3K-mTOR pathway [40]. To test which signaling pathways participate in PL-induced survivin expression, we used the MEK inhibitor PD 0325901 and the mTOR inhibitor rapamycin. Both of these inhibitors similarly prevented the PL-induced upregulation of survivin, implying that joint activation of both mTOR and MAPK pathway is needed for survivin upregulation. Importantly, these inhibitors did not have a major effect on *Tph1*, a known JAK2/STAT5 target. Collectively, these results allow to propose a signaling cascade illustrated in Fig 6C, highlighting the transactivation of EGFR as an important component in gestational beta-cell expansion triggered by Prl-R activation.

In summary, we provide the first 3D-volumetric analysis of the dynamics of β-cell mass expansion during murine pregnancy, showing that the number of islets nearly doubles during pregnancy. In addition, we show that EGFR signaling is involved in pregnancy- and lactogen-induced β-cell proliferation and that the mechanism involves survivin expression. Our findings further elucidate the physiological adaptation of β-cell mass to pregnancy.

## Supporting Information

Table S1 **List of primers used for RT-PCR.**
(PDF)

Movie S1 **Increased maternal β-cell mass during pregnancy.** Representative isosurface reconstructions from OPT-analyzed pancreata showing insulin in red and pancreatic tissue in light grey from virgin (control), pregnant gestational day 13.5 (GD13.5) and gestational day 18.5 (GD18.5) mice.
(MOV)
