# Supplementary material for: EGFR Signaling Promotes β-Cell Proliferation and Survivin Expression during Pregnancy
Source: PLoS One. 2014 Apr 2;9(4):e93651. doi: 10.1371/journal.pone.0093651 (PMC3973552; doi:10.1371/journal.pone.0093651)
Supplement: Table S1 — List of primers used for RT-PCR. (PDF) [file pone.0093651.s001.pdf]

| Primer | Forward sequence | Reverse sequence |
|---|---|---|
| **Birc5/Survivin (mouse)** | GACTGCAAAGACTACCCGTCA | GATGTGGCATGTCACTCAGG |
| **Tph1 (mouse)** | TCAAAAACTGGCAACGTGCTAC | GGCATGTCCAGAAAGTGCATG |
| **Tph2 (mouse)** | GTCAATTACCCGTCCCTTCTC | TCAATACTTCTGGTGTCTTTCAG |
| **EGFR (mouse)** | ATGACGCATTCCTCCCTGTA | TGATAATGCAGGTCTCTTCCA |
| **EGFR (human)** | GCGTGGACAAGTGCAACCTT | CTGAGGCAGGCACTCTGGG |
| **Htr2B (mouse)** | GGAGATATTTGTGTGGATAGG | TCCCGAAATGTCTTATTGAAGAG |
| **CyclophilinG (mouse)** | CAATGGCCAACAGAGGGAAG | CCAAAAACAACATGATGCCCA |
| **FoxM1 (mouse)** | ACAGCCTCACGATGAGAACC | TGATGTTTCACTCGGGGCAT |
| **Prl-R (mouse)** | TGCACTTGCTTACATGCTGC | CCTTGTCAGGGGAACGACAT |
